# The responses to long-term nitrogen addition of soil bacterial, fungal, and archaeal communities in a desert ecosystem

**DOI:** 10.3389/fmicb.2022.1015588

**Published:** 2022-10-13

**Authors:** Xuan Zhang, Xin Song, Taotao Wang, Lei Huang, Haiyang Ma, Mao Wang, Dunyan Tan

**Affiliations:** ^1^College of Life Sciences, Xinjiang Agricultural University, Ürümqi, China; ^2^State Key Laboratory of Grassland Agro-Ecosystems, Institute of Arid Agroecology, College of Ecology, Lanzhou University, Lanzhou, China

**Keywords:** nitrogen deposition, soil microbial community, desert ecosystem, bacteria, fungi, archaea

## Abstract

Nitrogen (N) deposition is a worldwide issue caused by human activity. Long-term deposition of N strongly influences plant productivity and community composition. However, it is still unclear how the microbial community responds to long-term N addition in a desert ecosystem. Therefore, a long-term experiment was conducted in the Gurbantonggut Desert in northwestern China in 2015. Four N addition rates, 0 (CK), 5 (N1), 20 (N2), and 80 (N3) kg N ha^−1^ yr.^−1^, were tested and the soil was sampled after 6 years of N addition. High-throughput sequencing (HTS) was used to analyze the soil microbial composition. The HTS results showed that N addition had no significant effect on the bacterial α-diversity and β-diversity (*p* > 0.05) but significantly reduced the archaeal β-diversity (*p* < 0.05). The fungal Chao1 and ACE indexes in the N2 treatment increased by 24.10 and 26.07%, respectively. In addition, N addition affected the bacterial and fungal community structures. For example, compared to CK, the relative abundance of Actinobacteria increased by 17.80%, and the relative abundance of Bacteroidetes was reduced by 44.46% under N3 treatment. Additionally, N addition also changed the bacterial and fungal community functions. The N3 treatment showed increased relative abundance of nitrate-reducing bacteria (27.06% higher than CK). The relative abundance of symbiotrophic fungi was increased in the N1 treatment (253.11% higher than CK). SOC and NH_4_^+^-N could explain 62% of the changes in the fungal community function. N addition can directly affect the bacterial community function or indirectly through NO_3_^−^-N. These results suggest that different microbial groups may have various responses to N addition. Compared with bacteria and fungi, the effect of N addition was less on the archaeal community. Meanwhile, N-mediated changes of the soil properties play an essential role in changes in the microbial community. The results in the present study provided a reliable basis for an understanding of how the microbial community in a desert ecosystem adapts to long-term N deposition.

## Introduction

Human activity, such as the use of fossil fuels and N fertilizer, increases the N deposition rate (Norby, 1998; Decina et al., 2020). From 1980 to 2018, ammonia emissions from global agricultural production increased by 78% (Liu L. et al., 2022). In China, the average soil N increased by 8 kg N ha^−1^ yr.^−1^ between the 1980s and 2000s (Liu et al., 2013). An increase in N deposition dramatically impacts plants and microorganisms (Fisher et al., 1988; Bobbink et al., 2010; Freedman et al., 2015). Moderate N deposition can alleviate N-limitation in a terrestrial ecosystem and promote primary productivity (Tipping et al., 2019), while excess N deposition leads to adverse effects on the ecosystem, such as soil acidification and heavy metal toxicity (Aber et al., 1998; Tian and Niu, 2015).

The desert ecosystem is an essential part of the terrestrial ecosystem, covering about one-third of the total land area (Laity, 2009). Because desert ecosystems are typically N-deficient, N has been considered as the primary factor affecting the structure and function of desert ecosystems (Brooks, 2003). In a desert ecosystem, N usually penetrates the soil in the form of pulses, accompanied by precipitation (e.g., snow) and other forms, which can constitute an adequate short-term supply of nutrition (Austin et al., 2004). The critical N load of a desert ecosystem is usually lower than that in other ecosystem types (Fenn et al., 2010). On the one hand, the plant cover in a desert ecosystem is usually sparse and discontinuous; therefore, the natural nutrient pools in deserts are small (Zhou et al., 2018). On the other hand, N is relatively limited in the soil, making a desert ecosystem particularly vulnerable to anthropogenic N input (Ladwig et al., 2012). Recent intensification of urbanization around deserts has caused reactive N to enter adjacent deserts by diffusion due to human activity, which increases the N deposition rate (Fenn et al., 2003). However, research on N deposition has mainly focused on forest and grassland ecosystems and less on desert ecosystems (Gilliam, 2006; Stursova et al., 2006), which significantly limits our ability to consider and predict the effects of N deposition across different ecosystems.

The duration of N deposition is an essential factor affecting the structure and function of the desert ecosystem, primarily the long-term accumulation of N in the environment (Humbert et al., 2016). Long-term N addition affects the ecosystem more dramatically than short-term addition (Kivimäki et al., 2013). Han et al. (2019) found that long-term N addition negatively affected plant diversity, but short-term N addition did not. In addition, the impact of N addition on an ecosystem may exhibit a time lag, and short-term N addition does not accurately reflect the impact of N addition on the ecosystem (Zhou et al., 2018). Therefore, long-term research is necessary to fully reveal the ecological effects of N deposition.

Soil microorganisms play an essential role in soil organic matter decomposition, plant fitness, and nutrient cycling (Berg and Matzner, 1997; Lau and Lennon, 2012; Ford et al., 2016). N addition can affect the microbial community (Lu et al., 2011; Ying et al., 2017), but different responses are seen in different ecosystems. N addition for 2 years changed the fungal community in a forest ecosystem (He et al., 2021). However, it did not significantly affect the microbial community in a grassland ecosystem (McHugh et al., 2017). In addition, distinct microbial types may have different responses to N addition. For example, N addition significantly affected the bacterial community structure but had little effect on the fungal community structure (Mueller et al., 2015). Currently, the researches on effects of N deposition are mainly focused on the bacterial and fungal communities and relatively less on the archaeal community. Archaea are a class of unicellular organisms that have similar morphology as bacteria, but show a close relationship with the fungi (Zhao et al., 2022). It has been found in most ecosystems and are indispensable in many ecological and biogeochemical processes (Meng et al., 2014). Accordingly, the study of archaea when exploring the response patterns of various microbial types in a desert ecosystem is necessary.

A long-term *in-situ* experiment was conducted at the Fukang Desert Ecosystem Observation and Experimental Station in 2015 to understand the long-term effects of N addition on soil microbial communities and their underlying mechanisms in a desert ecosystem. Soil samples were taken in mid-May 2021, and HTS of 16S rRNA gene and ITS were performed to study the effect of N deposition on the bacterial, fungal, and archaeal community structure and function. The objectives of this study were (1) to explore the response of soil microbial diversity, community structure, and function to N addition, and (2) to elucidate the factors driving changes in the soil microbial community when N is added.

## Materials and methods

### Study site description and experiment design

This study was conducted at the Fukang Desert Ecosystem Observation and Experimental Station, located in the southern periphery of the Gurbantonggut Desert, Northern Xinjiang, China (Supplementary Figures 1A,B). This area has a typical temperate continental climate. The annual mean temperature is 6.6°C, and the annual mean precipitation is 163 mm (Liu R. et al., 2022). The area has typical sandy soil with low nutrient status. The initial total N content of this soil was approximately 0.15 g kg^−1^ (Table 1), far lower than in a grassland or forest ecosystem (Zhang et al., 2014; Shi et al., 2018). The soil pH was 8–9 (Table 1), characterizing it as typical alkaline soil. The major plant community in this area includes shrubs and annual herbs, such as *Haloxylon ammodendron*, *H. persicum*, *Nepeta micrantha*, *Erodium oxyrhinchum*, and *Meniocus linifolius* (Huang et al., 2015; Yue et al., 2018).

**Table 1:** Effects of N addition on soil properties.

Treatment	pH	SOC (g kg^−1^)	TN (g kg^−1^)	NO_3_^−^-N (mg kg^−1^)	NH_4_^+^-N (mg kg^−1^)	AP (mg kg^−1^)
CK	8.74 ± 0.04a	2.94 ± 0.48b	0.15 ± 0.02a	2.96 ± 0.64b	2.22 ± 0.34b	7.18 ± 0.69a
N1	8.64 ± 0.14a	4.84 ± 1.03a	0.16 ± 0.02a	4.43 ± 0.61b	2.36 ± 0.23b	9.30 ± 1.86a
N2	8.48 ± 0.19a	3.38 ± 0.18ab	0.15 ± 0.02a	6.23 ± 1.21b	2.53 ± 0.17b	7.41 ± 0.67a
N3	8.46 ± 0.05a	3.21 ± 0.14ab	0.14 ± 0.02a	21.19 ± 3.15a	4.99 ± 0.05a	10.41 ± 0.57a

All data are presented as the mean ± standard error. SOC, soil organic carbon; TN, total nitrogen; NH_4_^+^-N, ammonium nitrogen; NO_3_^−^-N, nitrate nitrogen; AP, available phosphorus; CK, 0 kg N ha^−1^ yr.^−1^; N1, 5 kg N ha^−1^ yr.^−1^; N2, 20 kg N ha^−1^ yr.^−1^; N3, 80 kg N ha^−1^ yr.^−1^. Different lowercase letters in the same column indicate significance at *p* < 0.05.

The experimental plot was established in 2015 and adopted a completely randomized experimental design (Supplementary Figure 1C). The experimental area was maintained under natural conditions. Based on the actual atmospheric N deposition in this area (20–25 kg N ha^−1^ yr.^−1^, Zhang et al., 2012), four N treatments were established, including 0 (CK), 5 (N1), 20(N2), and 80 (N3) kg N ha^−1^ yr.^−1^. N2 treatment was approximately double the amount of atmospheric N deposition, while N1 and N3 treatments represented low and high N levels, respectively. Each treatment had three replicates. A total of twelve 2 m × 2 m quadrats were established and were bounded by a 0.5 m wide buffer strip. The form of N applied was NH_4_NO_3_ (35% pure N). The major plants in the sample plots were spring ephemerals that germinate in mid-late March and die in late May and early June (Huang et al., 2018). However, some Amaranthaceae plants continue their growth until October (Lu et al., 2013). Therefore, the added NH_4_NO_3_ was divided into six equal portions, and added to the plots during the plant growth season (i.e., April, May, and July to October) each year. The NH_4_NO_3_ was dissolved in 5 l of water and applied evenly with a sprayer. The same volume of water was supplied to the CK treatment to control for the effect of the water.

### Collection of soil samples and determination of soil properties

Soil samples were collected in the middle of May 2021. In this experiment, N addition continued until sampling. All plants within the sample plots were kept. The soil crust and plant litter were carefully removed from the soil surface during sampling. Soil samples were collected next to the plants at a depth of 0–5 cm using a three-point sampling method. Each soil sample was passed through a 2 mm mesh sieve, then divided into two parts, put into plastic self-sealing bags, packed into a refrigerated box, and transported to the laboratory. One subsample was used for measuring soil properties. The other subsample was stored at −20°C for the determination of the soil microbial community.

The soil pH was measured at a soil-to-water ratio of 1:5 with a pH meter (Seven Easy Plus, Shanghai, China; Rhoades, 1996). The soil organic carbon (SOC) was determined by the potassium dichromate external heating method (Ciavatta et al., 1991). The total nitrogen (TN) was determined by the Kjeldahl method (Bremner, 1965). The soil ammonium nitrogen (NH_4_^+^-N) and nitrate nitrogen (NO_3_^−^-N) were determined by indophenol blue colorimetry and ultraviolet spectrophotometry after extraction with 2 M KCl (Zhao et al., 2009). The available phosphorus (AP) was measured using the molybdenum antimony anti-colorimetry method (Zhang et al., 2015).

### DNA extraction and PCR amplification

Microbial DNA from the soil samples was extracted by the CTAB method (Berry et al., 2005). The concentration and purity of the DNA sample were determined by electrophoresis in 1% (w/v) agarose gels. The DNA samples were subsequently diluted to 1 ng/uL using sterile water. Polymerase chain reaction (PCR) amplification was conducted with the 515F (5′-GTGCCAGCMGCCGCGGTAA-3′) and 806R (5′-GGACTACHVGGGTWTCTAAT-3′) primers, which amplified the hypervariable regions V3–V4 of the bacterial 16S rRNA gene (Pichler et al., 2018). The primers ITS5-1737F (5′-GGAAGTAAAAGTCGTAACAAGG-3′) and ITS2-2043R (5′-GCTGCGTTCTTCATCGATGC-3′) were used to amplify the fungal ITS1 region (Mukhtar et al., 2021). The hypervariable V4 regions of the archaeal 16S rRNA gene were amplified with the primer sets Arch519F (5′-CAGCCGCCGCGGTAA-3′) and Arch915R (5′-GTGCTCCCCCGCCAATTCCT-3′) (Li et al., 2022). All PCR reactions were carried out in a 30 μl reaction system with 15 μl of Phusion® High-Fidelity PCR Master Mix (BioLabs, Ipswich, MA, United States): approximately10 ng of template DNA, and 0.2 μM of forward and reverse primers. Thermal cycling conditions were as follows: initial denaturation at 98°C for 1 min, followed by 30 cycles of denaturation at 98°C for 10 s, annealing at 50°C for 30 s, and elongation at 72°C for 30 s. Finally, 72°C for 5 min. The PCR products were visualized by agarose gel electrophoresis and were mixed in equidensity ratios. Then, the mixture of PCR products was purified with a Gene JET Gel Extraction Kit (Thermo Fisher Scientific). Sequencing libraries were generated using the Illumina TruSeq DNA PCR-Free Library Preparation Kit following the manufacturer’s recommendations, and index codes were added. The library quality was assessed on the Qubit@ 2.0 Fluorometer (Thermo Fisher Scientific) and Agilent Bioanalyzer 2,100 system. Finally, the library was created by HTS on the Illumina NovaSeq 6,000 platform at Novogene (Beijing, China). All the sequences of bacterial and archaeal 16S rRNA, and of fungal ITS have been saved in the SRA of the National Center for Biotechnology Information under accession number PRJNA863934.

### Bioinformatics analysis

Paired-end reads from the original DNA fragments were spliced using the FLASH analysis tool (version 1.2.7^1^; Magoč and Salzberg, 2011), and the obtained splicing sequence comprised the Raw tags. Raw tags obtained by splicing suffered strict filtering to produce high-quality Clean Tags. The QIIME software (version 1.9.1^2^; Caporaso et al., 2010) was used to remove the chimeric sequence, and to obtain the Effective Tags. Using the Uparse algorithm (version 7.0.1001^3^) to cluster all effective tags of all samples, sequences with ≥97% similarity were assigned to the same OTU. For bacteria and archaea, the Silva Database (version 138.1^4^) based on the Mothur algorithm was used to annotate taxonomic information. Fungal species annotation was performed by using the blast algorithm in the QIIME software (version 1.9.1^5^) and the Unite database (version 8.2^6^). The Chao1 and ACE richness and the Shannon and Simpson diversity indices were calculated to measure microbial α-diversity using QIIME software (version 1.9.1). Bacterial and archaeal community functions in the ecological samples were predicted according to the FAPROTAX database (Sansupa et al., 2021), while the fungal community function was predicted according to the FUNGuild database (Nguyen et al., 2016).

### Statistical analysis

One-way analysis of variance (ANOVA) with the least significant difference (LSD) was used to analyze the significant differences among N addition treatments for soil properties and the microbial community with the SPSS 22.0 software package (SPSS Inc., Chicago, IL, United States). Before the analysis, these data were transformed as necessary to meet the requirements of variance homogeneity and normal distribution. If the data were still not satisfied after conversion, the non-parametric test was carried out.

A Venn diagram was used to analyze the differences between shared and unique OTU numbers among the four N treatments. A principal co-ordinates analysis (PCoA) based on Bray–Curtis dissimilarity was constructed to study the differences between the four treatments using the vegan software package (version 2.15.3; Freedman and Zak, 2014). The microbial β-diversity index was calculated using the QIIME software (version 1.9.1), and pairwise differences were further compared based on the Wilcoxon rank sum test. A LEfSe analysis (LDA score ≥ 2.0) was used to determine the significant differences in microbial community from the phylum to the genus levels using the LEfSe software (version 1.0; Qin et al., 2021). The soil properties as explanatory variables and the dominant microbial phyla as species variables were used to perform a redundancy analysis (RDA) with the vegan packages in the R software (version 2.15.3). A Spearman correlation analysis was used to further explore the relationships between the soil properties and the dominant microbial phyla. A Mantel-test analysis based on the OTU level was used to describe the relationship between the soil properties and the microbial community structure. Structural equation modeling (SEM) was used to understand how the soil properties mediate the microbial community richness and function changes using AMOS 26.0. The microbial community richness was represented by the ACE index, which has a higher fitness than the Chao1 index. All functional groups of soil bacteria, fungi, and archaea were ranked using a principal component analysis (PCA), and the first principal component (PC1) was used in an SEM analysis. The fitness of the SEM was assessed using the Chi-square (χ2) test, the comparative fit index (CFI > 0.9), and the root mean square error of approximation (RMSEA <0.08; Zhao et al., 2020).

## Results

### Effect of N addition on soil properties

N addition did not significantly affect the pH, TN, and AP (*p* > 0.05) but significantly increased the content of SOC, NH_4_^+^-N, and NO_3_^−^-N (*p* < 0.05; Table 1). Compared with CK, the SOC content of the N1 treatment significantly increased by 64.63% (*p* < 0.05). The N3 treatment significantly increased the content of NH_4_^+^-N and NO_3_^−^-N by 124.77 and 615.87%, respectively (*p* < 0.001).

### Effects of N addition on bacterial, fungal, and archaeal diversities and community structures

**Figure 1:**
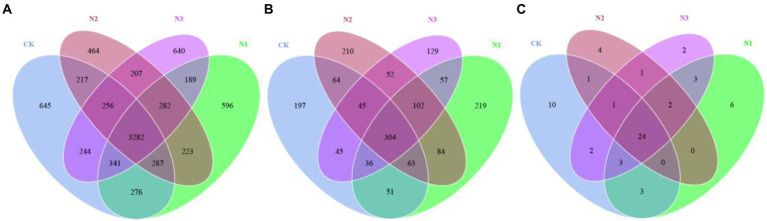
Venn analysis of the soil microbial community in different N deposition treatments based on OTU levels. **(A)** The number of bacterial OTU at different N treatments. **(B)** The number of fungal OTU at different N treatments. **(C)** The number of archaeal OTU at different N treatments. Each ellipse represents a treatment in the figure, and the number of shared OTUs between treatments is represented by the number of overlapping parts, while the number of non-overlapping parts represents the number of unique OTUs between treatments.

**Figure 2:**
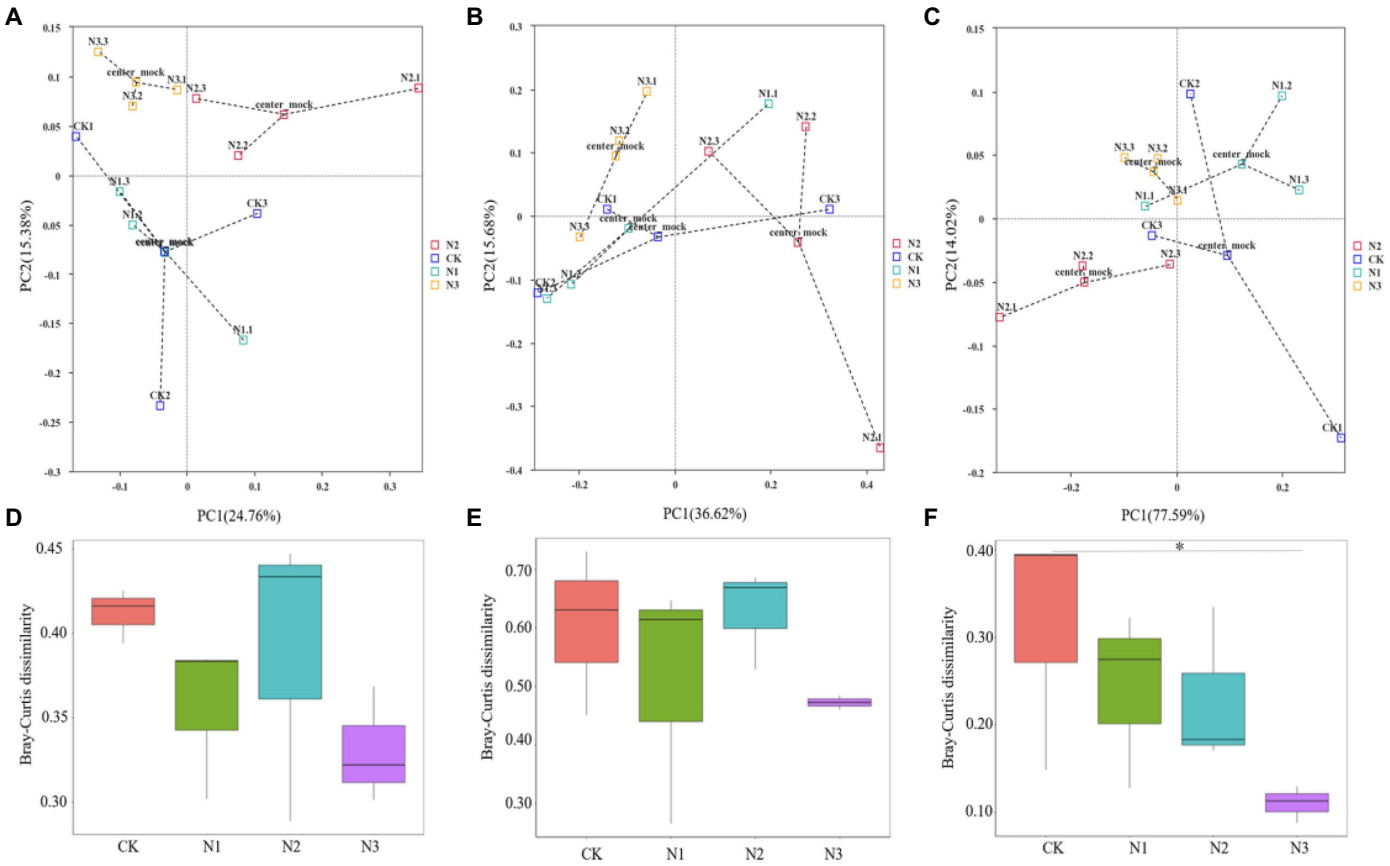
Effects of N addition on soil microbial community β-diversity revealed by PCoA analysis **(A–C)** and Wilcoxon rank sum test **(D–F)**. **(A,D)** Bacterial community. **(B,E)** Fungal community. **(C,F)** Archaeal community. In **(A–C)**, each point represents a sample, and points of the same color come from the same treatment. The closer the distance between two points is, the smaller the difference exists in community composition between them. The center mock represents the center point of the same sample confidence circle. * Indicates *p* < 0.05.

Among the four N treatments, bacteria, fungi, and archaea, respectively, shared 3,282, 304, and 24 OTUs (Figure 1). All OTUs were used in the subsequent statistical analyses. N addition did not significantly affect the bacterial and archaeal OTU and the α-diversity indexes (i.e., Shannon, Simpson, Chao1, and ACE), but the OTU, Chao1, and ACE indexes of the fungi were significantly increased (*p* < 0.05; Table 2). Compared to CK, the OTU and Chao1 index of fungi in the N2 treatment increased by 24.72 and 24.10%, respectively. The ACE index of fungi in the N1 and N2 treatments increased by 23.73 and 26.07%, respectively. A PCoA analysis based on the Bray–Curtis dissimilarity showed that the PC1 and PC2 dimensions explained 24.76 and 15.38% of the OTU information for the soil bacterial community (Figure 2A), 36.62 and 15.68% of the OTU information for the soil fungal community (Figure 2B), and 77.59 and 14.02% of the OTU information for the soil archaeal community (Figure 2C). The degree of dispersion between treatments in the PCoA analysis represents the difference between treatments, the more dispersed between treatments, the more significant differences in microbial communities. The PCoA analysis revealed that the N addition could affect the microbial community, but different microbial groups presented distinct responses (Figures 2A–C). Compared with fungi and archaea, the bacterial community was more sensitive to N addition since the bacterial communities in N2 and N3 treatments were separated from each other and from that in CK and N1 treatments. However, the communities of fungi and archaea in N2 and N3 treatments were separated from each other but not from that of CK and N1 treatments. Comparing the pairwise differences based on the Wilcoxon rank sum test showed that N addition did not significantly affect the bacterial and fungal β-diversity, but archaeal β-diversity was significantly reduced in the N3 treatment compared to CK (*p* < 0.05; Figures 2D–F).

**Table 2:** Effects of N addition on soil microbial OTUs and the α-diversity indices.

Microbial species		OTU	Shannon index	Simpson index	Chao1 index	ACE index
Bacteria	CK	3306.33 ± 64.43a	9.66 ± 0.18a	0.9942 ± 0.0024a	3356.27 ± 61.85a	3412.48 ± 47.62a
	N1	3303.00 ± 75.06a	9.50 ± 0.06a	0.9934 ± 0.0010a	3341.75 ± 72.79a	3400.33 ± 67.51a
	N2	3197.33 ± 150.51a	9.67 ± 0.23a	0.9953 ± 0.0016a	3228.85 ± 161.96a	3268.15 ± 159.76a
	N3	3385.67 ± 66.61a	9.80 ± 0.06a	0.9963 ± 0.0003a	3403.61 ± 54.46a	3452.98 ± 43.04a
Fungi	CK	392.33 ± 31.32b	3.79 ± 0.63a	0.7473 ± 0.1033a	396.56 ± 27.64b	400.90 ± 29.12c
	N1	474.00 ± 8.89ab	3.60 ± 0.71a	0.6658 ± 0.1336a	472.81 ± 8.56ab	496.02 ± 5.60ab
	N2	489.33 ± 31.39a	4.86 ± 0.15a	0.8984 ± 0.0205a	492.15 ± 37.92a	505.42 ± 31.92a
	N3	408.33 ± 22.24ab	3.59 ± 0.25a	0.7572 ± 0.0527a	413.09 ± 21.97ab	425.73 ± 20.38bc
Archaea	CK	27.67 ± 1.67a	2.58 ± 0.24a	0.7699 ± 0.0439a	28.33 ± 2.40a	29.41 ± 3.27a
	N1	28.33 ± 1.20a	2.60 ± 0.13a	0.7770 ± 0.0329a	28.88 ± 0.83a	29.08 ± 1.44a
	N2	21.67 ± 3.93a	1.98 ± 0.33a	0.5960 ± 0.0950a	21.79 ± 4.39a	25.03 ± 2.19a
	N3	24.33 ± 3.28a	2.33 ± 0.15a	0.7237 ± 0.0293a	23.82 ± 3.34a	25.80 ± 4.60a

All data are presented as the mean ± standard error. OTU, operational taxonomic unit. CK, 0 kg N ha^−1^ yr.^−1^; N1, 5 kg N ha^−1^ yr.^−1^; N2, 20 kg N ha^−1^ yr.^−1^; N3, 80 kg N ha^−1^ yr.^−1^. Different lowercase letters in the same column indicate significance at *p* < 0.05.

**Figure 3:**
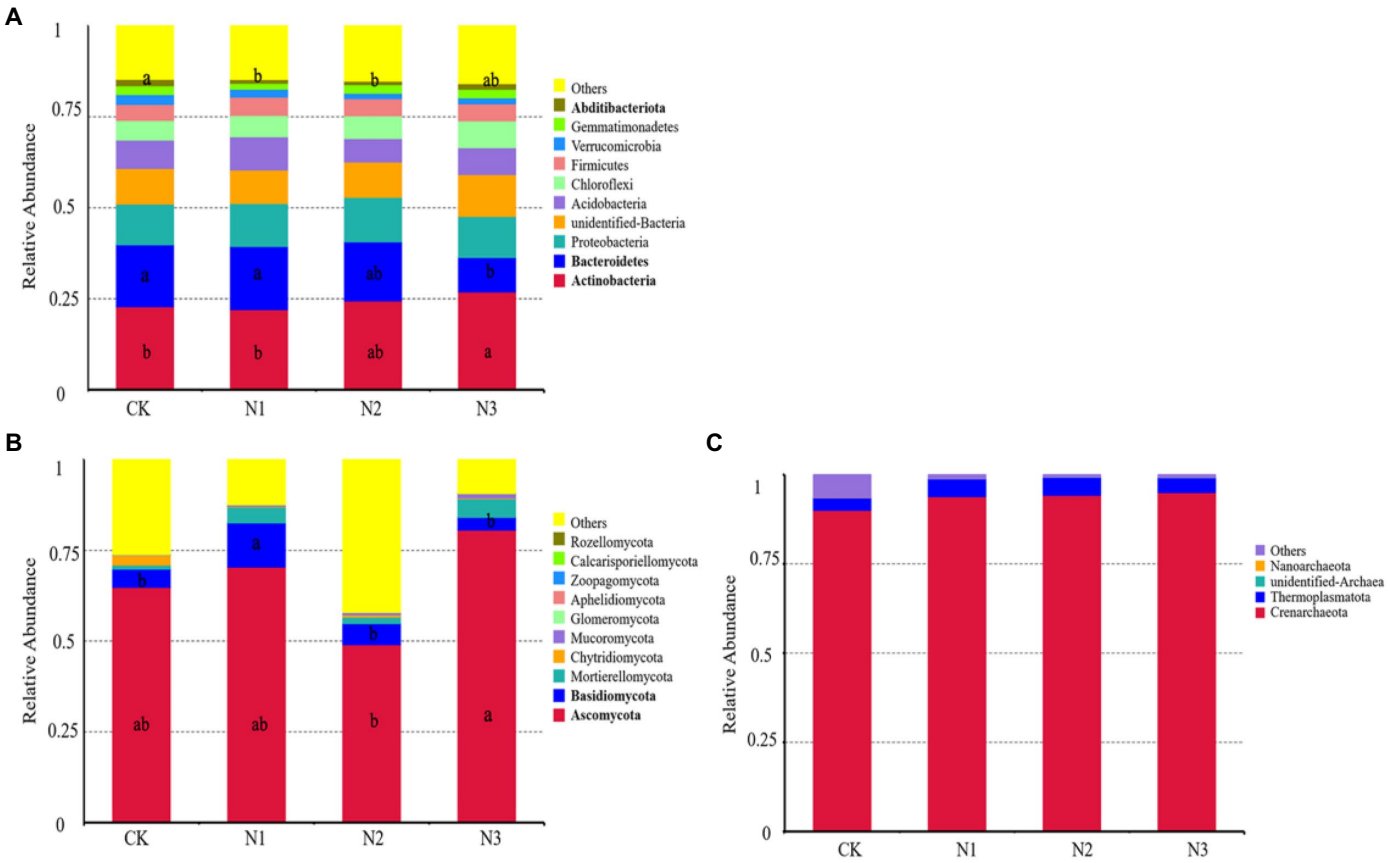
Effect of N addition on the soil microbial community (phylum level). **(A)** The relative abundance of the top 10 bacterial phyla. **(B)** The relative abundance of the top 10 fungal phyla. **(C)** The relative abundance of the archaeal phyla.

The relative abundance of the top 10 phyla of bacteria, fungi, and archaea was selected to evaluate the effect of N addition on soil microbial community structure (Figure 3). Actinobacteria (CK: 22.84%, N1: 22.02%, N2: 24.43%, N3: 26.91%), Bacteroidetes (CK: 17.05%, N1: 17.37%, N2: 16.22%, N3: 9.47%) and Proteobacteria (CK: 11.15%, N1: 11.84%, N2: 12.22%, N3: 11.28%) were the dominant bacterial phyla (Figure 3A). Compared with CK, the high N addition (N3 treatment) significantly increased the relative abundance of Actinobacteria by 17.80%, while significantly decreased the relative abundance of Bacteroidetes by 44.46% (p < 0.05). In addition, N addition presented a trend to increase the average relative abundance of Chloroflexi (CK: 5.39%, N1: 5.88%, N2: 6.28%, N3: 7.37%) and to decrease the average relative abundance of Verrucomicrobia (CK: 2.75%, N1: 2.15%, N2: 1.52%, N3: 1.63%). The relative abundance of Ascomycota was the highest among the fungal phyla (Figure 3B). Low level of N addition (N1 treatment) significantly increased the relative abundance of Basidiomycota, compared with CK, by 141.67% (*p* < 0.05). Moreover, N addition increased the average relative abundance of Mucoromycota (CK: 0.09%, N1: 0.43%, N2: 0.64%, N3: 1.19%), and decreased the average relative abundance of Chytridiomycota (CK: 2.74%, N1: 0.21%, N2: 0.62%, N3: 0.22%). A total of four archaeal phyla were detected in all the treatments (Figure 3C). The abundance of Crenarchaeota was the highest among the archaeal phyla (CK: 90.04%, N1: 93.87%, N2: 94.18%, N3: 95.01%). N addition increased the average relative abundance of Thermoplasmatota.

**Figure 4:**
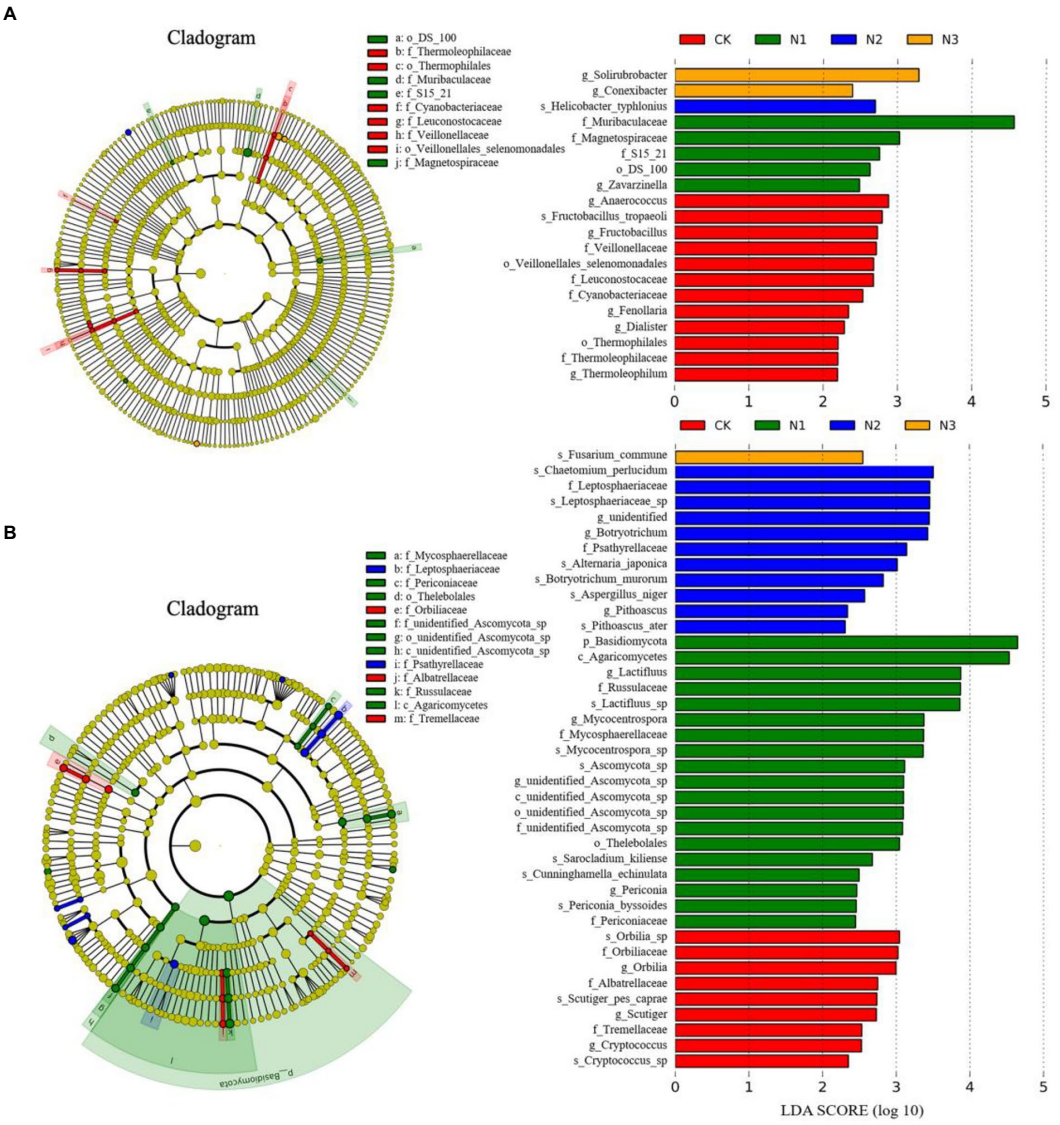
The LEfSe analysis of the soil microbial community structure regarding N addition (phylum-genus levels). **(A)** The LEfSe analysis of the bacterial community (LDA ≥ 2.0). **(B)** The LEfSe analysis of the fungal community (LDA ≥ 2.0).

A LEfSE analysis was performed between treatments to identify the taxa (i.e., biomarkers) with significant differences in abundance (Figure 4). With an LDA threshold of 2, the LEfSe results showed 20 biomarkers in the bacterial community (Figure 4A): 12 in CK (including Thermophilales, Cyanobacteriaceae, *Anaerococcus*, and *Fructobacillus*), 5 in N1 treatment (including Muribaculaceae and Magnetospiraceae), 1 in N2 treatment (*Helicobacter typhlonius*), and 2 in N3 treatment (*Solirubrobacter* and *Conexibacter*). The LEfSe identified 40 biomarkers in the fungal community (Figure 4B): 9 in CK treatment, mainly including Orbiliaceae, Tremellaceae, Albatrellaceae, and *Cryptococcus*; 19 in N1 treatment (mainly the Basidiomycota taxa); 11 in N2 treatment, mainly including Leptosphaeriaceae and Psathyrellaceae; 1 in N3 treatment (*Fusarium commune*). No biomarker was found in the archaeal community in any treatment.

### Effects of N addition on bacterial, fungal, and archaeal community functions

**Figure 5:**
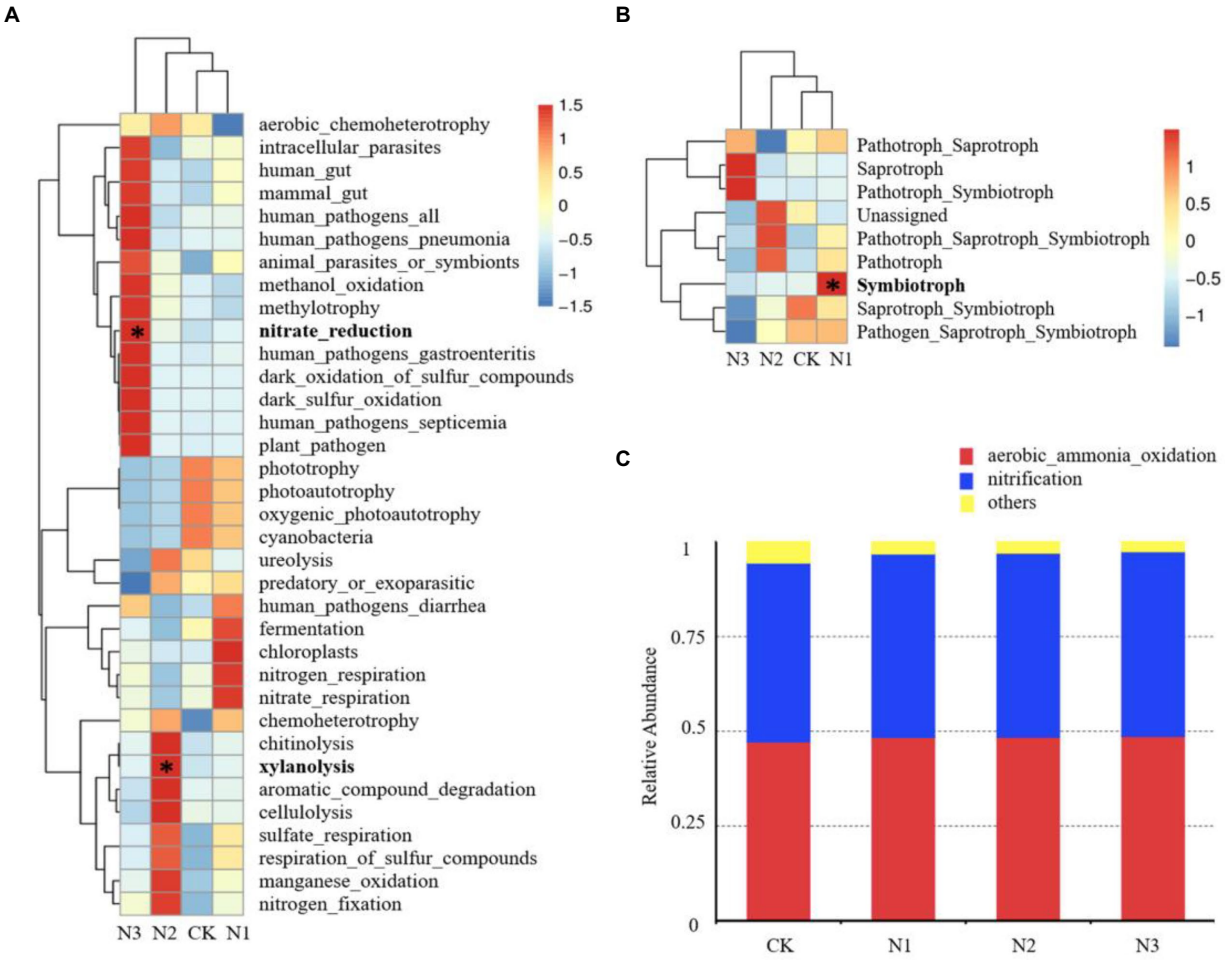
Effect of N addition on microbial community functions. **(A)** Effect of N addition on the relative abundance of the top 35 bacterial community functions. **(B)** Effect of N addition on the relative abundance of fungal community functions. **(C)** Effect of N addition on the relative abundance of archaeal community functions. * Indicates *p* < 0.05.

According to the species classification results, 35 bacterial functions with a high relative abundance were selected for study (Figure 5A). The relative abundance of chemoheterotrophy (14.73–15.40%), aerobic chemoheterotrophy (13.09–14.33%), nitrate reduction (6.44–8.07%), and fermentation (1.64–2.83%) were high. The relative abundance of nitrate-reducing bacteria was significantly increased under N3 treatment (27.06%, *p* < 0.05) compared with that in CK. Additionally, the relative abundance of xylanolytic bacteria was significantly increased by 162.94% in N2 treatment (*p* < 0.05). Based on the annotated FUNGuild database, the fungal functional community showed 9 nutritional patterns, and pathotroph-saprotroph (26.96–56.87%) was the main fungal function (Figure 5B). The abundance of symbiotrophic fungi was significantly increased under N1 treatment (253.11%, *p* < 0.05) compared with that in CK. In addition, the average relative abundance of saprotrophic fungi was increased in N3 treatment. The archaeal functional community was annotated according to the FAPROTAX database. Two archaeal functions were detected, including aerobic ammonia oxidation and nitrification, and N addition did not affect their relative abundance (Figure 5C).

### Relationship between bacterial, fungal, and archaeal community structures and soil properties

**Figure 6:**
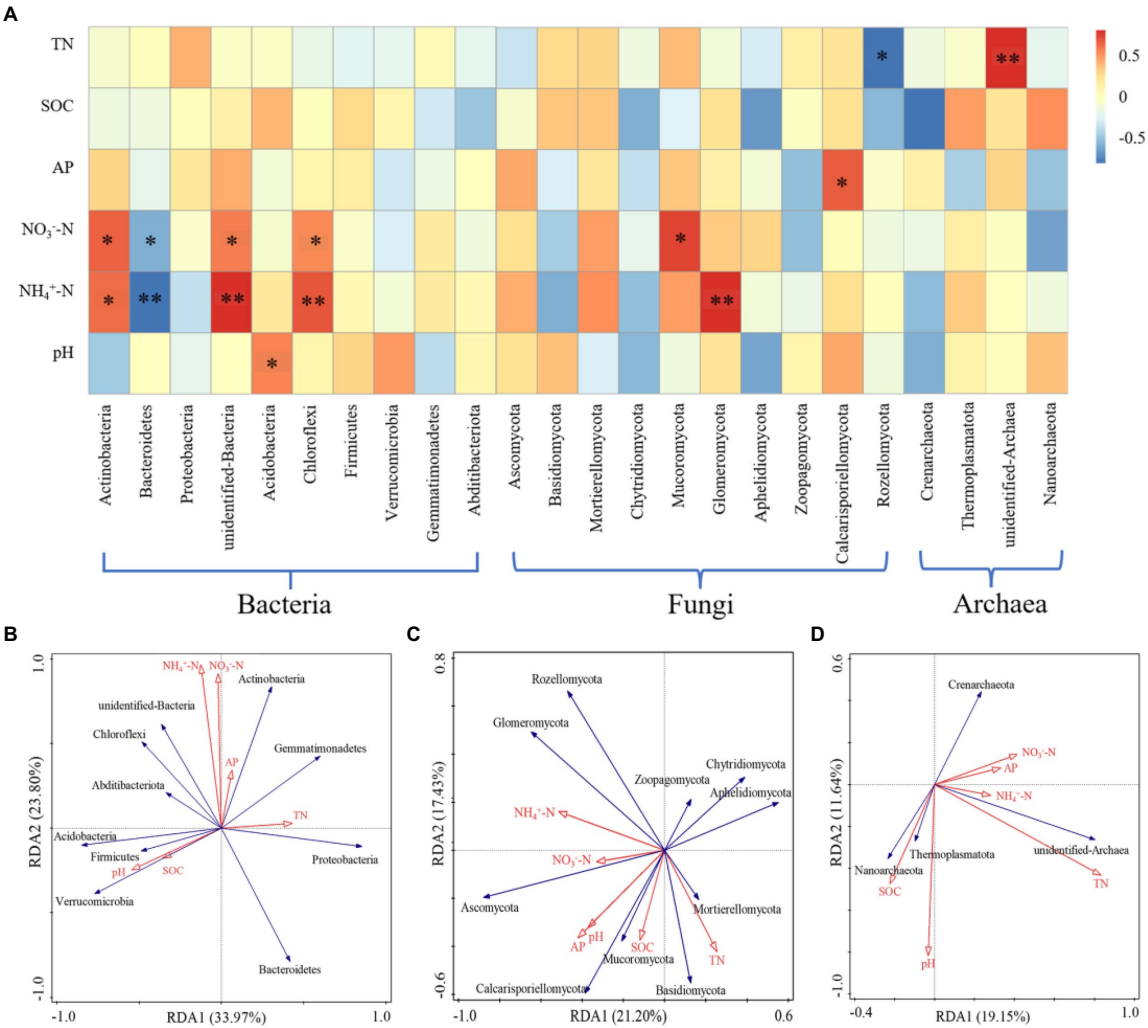
The analysis of the relationship between microbial community structure and soil properties (phylum level). **(A)** Spearman correlation analysis of microorganisms. **(B)** RDA analysis of bacteria. **(C)** RDA analysis of fungi. **(D)** RDA analysis of archaea. * Indicates *p* < 0.05, ** indicates *p* < 0.01.

The relationship between the microbial community structure and soil properties at the phylum level was analyzed by RDA and Spearman analyses (Figure 6). The soil factors could explain 57.77% of the bacterial community variation; RDA1 explained 33.97% of the variation, and RDA2 explained 23.80% of the variation (Figure 6B). The concentrations of NH_4_^+^-N and NO_3_^−^-N were significantly positively correlated with Actinobacteria, unidentified bacteria, and Chloroflexi, but they were negatively correlated with Bacteroidetes. The pH was significantly positively correlated with Acidobacteria (*p* < 0.05; Figure 6A). The soil factors could explain 38.63% of the total variation of the fungal community; RDA1 explained 21.20% of the variation, and RDA2 explained 17.43% of the variation (Figure 6C). Glomeromycota was significantly positively correlated with NH_4_^+^-N (*p* < 0.01). NO_3_^−^-N was significantly positively correlated with Mucoromycota. AP was significantly positively correlated with Calcarisporiellomycota. TN was significantly negatively correlated with Rozellomycota (*p* < 0.05; Figure 6A). The soil factors could explain 30.79% of the total variation of the archaeal community; RDA1 explained 19.15% of the variation, and RDA2 explained 11.64% of the variation (Figure 6D). TN was significantly positively correlated with unidentified archaea (*p* < 0.01; Figure 6A).

## Discussion

### Effects of N addition on soil properties

In the present study, soil pH, TN content, and AP content were not significantly affected by the addition of N, meanwhile the contents of SOC, NH_4_^+^-N, and NO_3_^−^-N seemed N level dependent features, since the content of SOC was only significantly increased in the N1 treatment, and the contents of NH_4_^+^-N and NO_3_^−^-N were only significantly increased in the N3 treatment (Table 1). The increase of SOC might be related to the possibly enhanced input of plant residue (Sha et al., 2021), the relatively high abundance of photosynthetic bacteria, and the relatively low abundance of degradation bacteria and saprotrophic fungi in the N1 treatment (Figures 5A,B). The increase in contents of NH_4_^+^-N and NO_3_^−^-N by the high level of N addition (N3 treatment) was consistent with previous studies (Zheng et al., 2021). Although NH_4_^+^-N and NO_3_^−^-N were added at equimolar concentrations, their accumulation rates were different (NO_3_^−^ > NH_4_^+^), similar with the observation of Huang et al. (2021) in the desert steppe. The NH_4_^+^-N and NO_3_^−^-N accumulation rates may be related to the alkaline soil (pH = 8.74; Table 1) in this study, since acidic soils favored the uptake of NO_3_^−^-N by plants, while alkaline soil favored the uptake of NH_4_^+^-N (Marcus-Wyner, 1983). The absence of change in TN content in treatments of N addition in this study might be explained by the assimilation of plants (Cui et al., 2019) and the emission of gases such as NH_3_ and N_2_O produced by nitrate respiration (Yue et al., 2019).

### Effects of N addition on soil microbial diversity and community structure

**Figure 7:**
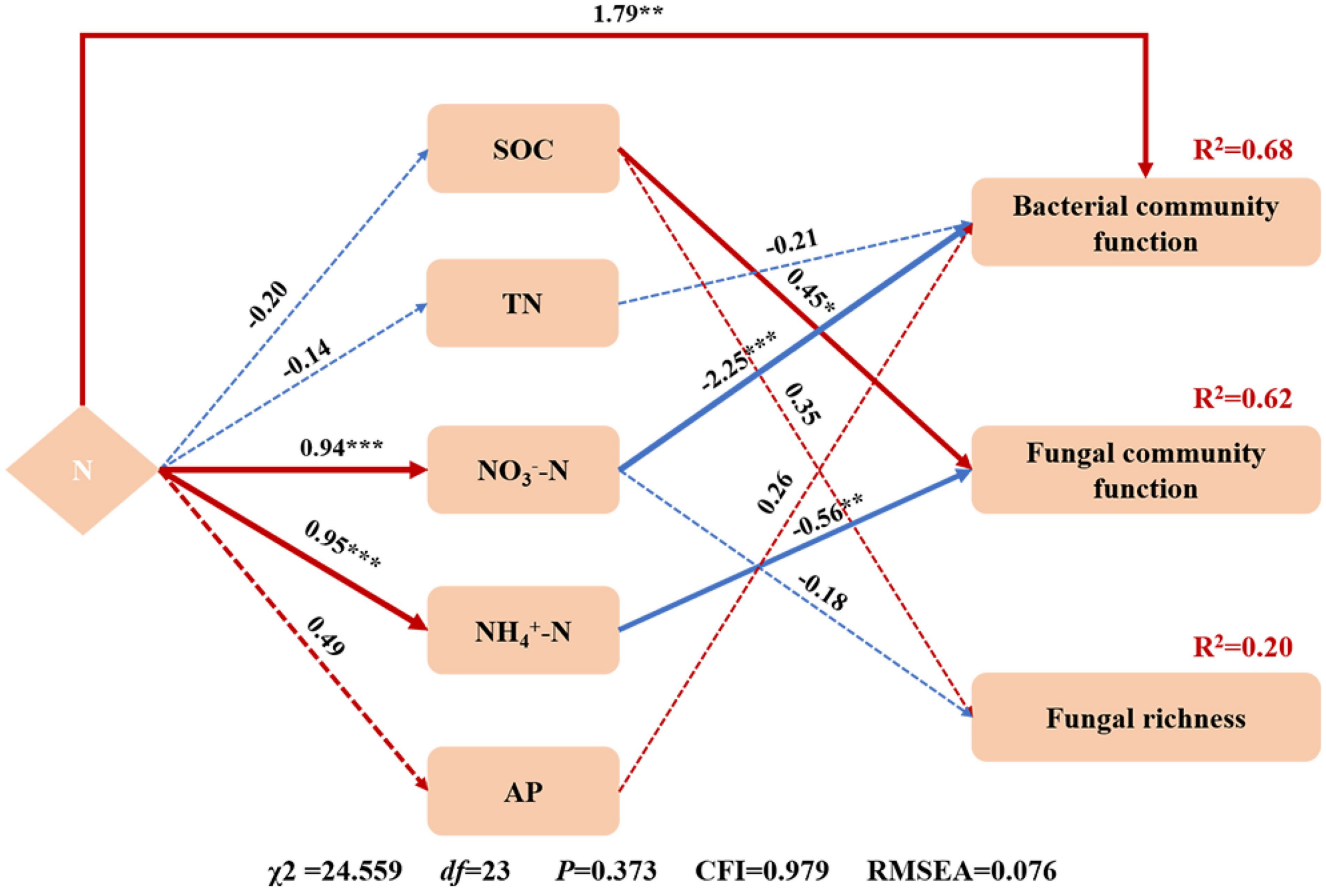
Structural equation model (SEM) of the effects of N addition on soil properties (SOC, TN, NO_3_^−^-N, NH_4_^+^-N, and AP) and microbial community richness and function. Arrows indicate the hypothesized direction of causation. The numbers next to the arrows are standardized path coefficients. The width of the arrows represents the strength of the relationships. Red arrows represent positive and blue arrows represent negative relationships. The solid arrows represent significant, and the dashed arrows represent non-significant relationships. To improve the adaptability of the model, delete the paths with less correlation. * Indicates *p* < 0.05, ** indicates *p* < 0.01, *** indicates *p* < 0.001.

In this study, N addition did not affect the Shannon and Simpson indices of the soil microbes, which is consistent with previous studies (Yuan et al., 2020), but contrasted with the results obtained from a forest ecosystem (Liu Y. et al., 2022), in which 6 years of N addition reduced the microbial diversity. This might be related to the fact that the desert ecosystem differs from the forest ecosystem and their soil microorganisms have different sensitivity to N addition (Ramirez et al., 2010). N addition did not affect bacterial and archaeal richness but significantly increased fungal richness in the N2 treatment (Table 2), which might be dependent on the initial soil conditions (Wang et al., 2018). The fungal community richness increased with N at nutrient-poor sites but decreased at nutrient-rich sites (Moore et al., 2021). The SEM showed that SOC and NO_3_^−^-N could explain 20% of the changes in fungal richness (Figure 7). In addition, the variation among bacterial, fungal, and archaeal richness responses to N addition might be related to the demand and physiological structure of microorganisms themselves. Soil bacteria were mainly affected by predation rather than resource effectiveness (Wardle et al., 1995), while the fungal community was sensitive to changes in resource effectiveness associated with N deposition (Allison et al., 2007; Wang et al., 2021a). N addition did not affect bacterial and fungal β-diversity, which is consistent with Liu et al. (2021). In addition, N addition significantly reduced the archaeal β-diversity in the N3 treatment (Figure 2F), indicating that the N addition makes the archaeal community more convergent.

N addition changed the bacterial and fungal community structures at the phylum level and had little effect on the archaeal community structure (Figure 3). A LEfSe analysis also confirmed this result (Figure 4). The relative abundances of Actinobacteria, Ascomycota, and Crenarchaeota were the highest among the phyla in bacteria, fungi, and archaea, respectively. N addition increased the relative abundances of the phyla Actinobacteria, Chloroflexi, and Mucoromycota, but decreased the relative abundances of Bacteroidetes and Verrucomicrobia. The co-trophic hypothesis could explain these results that nutrient-rich soil is conducive to the growth of copiotrophic taxa, with oligotrophic taxa exhibiting an opposite pattern (Fierer et al., 2012; Grant et al., 2022). In this study, N addition increased the relative abundance of copiotrophic taxa, such as Actinobacteria, and decreased the relative abundance of oligotrophic taxa, such as Verrucomicrobia.

Based upon the data in Supplementary Table 1, only the pH was significantly correlated with the community structure of fungi and archaea at the OTU level in the tested soil, while the other soil traits, including SOC, TN, NO_3_^−^-N, NH_4_^+^-N, and AP, had no significant effects on the community structure of bacteria, fungi, and archaea at the OTU level. This is consistent with previous studies that soil pH was the critical factor that affected the microbial community structure (Francioli et al., 2016; Ullah et al., 2019; Zheng et al., 2019).

### Effects of N addition on soil microbial community function

Long-term N addition affected the bacterial and fungal community functions but did not affect the archaeal function (Figure 5). These effects were consistent with the response pattern of the microbial community structure. In this study, high level N addition (N3 treatment) significantly increased the relative abundance of the nitrate-reducing bacteria (Figure 5A), which are positively correlated with NO_3_^−^-N (Yang et al., 2017). The input of NH_4_NO_3_ increased the content of NO_3_^−^-N in soil (Table 1), thereby promoting the abundance of nitrate-reducing bacteria (Mohn et al., 2000). In addition, N2 treatment, but not N1 and N3 treatments, significantly increased the relative abundance of the xylanolytic bacteria (Figure 5A). It seems that this increase is not related to the SOC (Table 1), but possible related to the C/N ratio in soil. Xylan is the main component of hemicellulose, accounting for 20–30% of the total hemicellulose (Gírio et al., 2010). Cellulose and hemicellulose are the main components of the plant cell wall. The increased relative abundance of xylanolytic bacteria can promote the decomposition of plants. This result is consistent with the promotion of litter decomposition upon N addition (Hou et al., 2021). At the same time, N addition also increased the average relative abundance of saprotrophic fungi (Figure 5B). Saprotrophic fungi play an essential role in decomposition because they can attack the lignocellulose matrix in the litter (Adl, 2003). The relative abundance of saprotrophic fungi in the N3 treatment (81.17% higher than CK) further confirmed that N addition promoted plant litter decomposition. Mucoromycota is saprotrophic fungi, and can decompose organic matter (Rashmi et al., 2019). In this study, the average relative abundance increases of Mucoromycota may be an essential factor for the increase of the relative abundance of the saprotrophic fungi in general. The relative abundance of the symbiotrophic fungi increased significantly in the N1 treatment (Figure 5B), contrary to previous studies (van Diepen et al., 2010; Cheng et al., 2020). The relative abundance of symbiotrophic fungi was positively correlated with the carbon input (Högberg et al., 2010). The content of SOC increased significantly in the N1 treatment (Table 1), promoting symbiotrophic fungal growth. N addition had no effect on aerobic ammonia oxidizing archaea (Figure 5C), which is consistent with Long et al. (2012). N addition can change the abundance of aerobic ammonia oxidizing archaea in acidic soil (Isobe et al., 2012) but had no effect in alkaline soil (Shen et al., 2008). The experimental area soil is typically alkaline, with a pH of 8.74 (Table 1). Therefore, N addition did not affect the abundance of aerobic ammonia oxidizing archaea.

Soil properties are key factors affecting the microbial community. The SEM analysis explained the bacterial and fungal community function changes (Figure 7). N addition, TN, NO_3_^−^-N, and AP can explain 68% of the changes in the bacterial community function. N addition can directly affect bacterial community function. At the same time, it can also indirectly affect bacterial community function by changing the soil properties. In this study, NO_3_^−^-N is a vital soil factor affecting bacterial community function. The change in the fungal community function is mainly due to soil properties. SOC and NH_4_^+^-N can explain 62% of the changes in the fungal community function. SOC had a significant positive effect on the fungal community function, and NH_4_^+^-N significantly negatively impacted the fungal community function. The effect of SOC on the fungal community function may primarily promote the physiological metabolism of soil microorganisms through labile organic carbon (Cleveland et al., 2007; Wang et al., 2021b). N addition changed the content of SOC (Table 1), thus affecting the fungi community function.

## Conclusion

In conclusion, N addition in the tested desert region presented different effects on the community structures and functions of bacteria, fungi, and archaea, respectively. Long-term (6 years) of N addition did not affect bacterial α-diversity and β-diversity. The fungal richness was significantly increased in the N2 (20 kg N ha^−1^ yr.^−1^) treatment. Although N addition did not affect the archaeal α-diversity, the β-diversity was significantly reduced in the N3 (80 kg N ha^−1^ yr.^−1^) treatment. In addition, N addition affects the bacterial and fungal community structures. Soil pH was significantly correlated with the fungal and archaeal community structures at the OTU level. N addition also changed bacterial and fungal community functions, directly or indirectly through NO_3_^−^-N for bacteria and *via* SOC and NH_4_^+^-N for fungi. Our study mainly focused on a desert ecosystem, but the effect of N deposition is highly environment-dependent and scale-dependent. To fully elucidate the effect of N deposition, further studies are necessary to carry out research on a regional and global scale.

## Data availability statement

The datasets presented in this study can be found in online repositories. The names of the repository/repositories and accession number(s) can be found at: https://www.ncbi.nlm.nih.gov/, PRJNA863934.

## Author contributions

XZ and MW designed the research and wrote the manuscript. XZ and XS analyzed the data. XZ, HM, TW, and LH were responsible for performing the fieldwork. All authors contributed to the article and approved the submitted version.

## Funding

This study was supported by the National Natural Science Foundation of China (No. 31960260), Tianshan Youth Program of Xinjiang Uygur Autonomous Region (No. 2019Q078) and the Key Research and Development Program of the Xinjiang Uygur Autonomous Region (No. 2022B02003).

## Conflict of interest

The authors declare that the research was conducted in the absence of any commercial or financial relationships that could be construed as a potential conflict of interest.

## Publisher’s note

All claims expressed in this article are solely those of the authors and do not necessarily represent those of their affiliated organizations, or those of the publisher, the editors and the reviewers. Any product that may be evaluated in this article, or claim that may be made by its manufacturer, is not guaranteed or endorsed by the publisher.

## Footnotes

^1^ http://ccb.jhu.edu/software/FLASH/

^2^ http://qiime.org/scripts/split_libraries_fastq.html

^3^ http://www.drive5.com/uparse/

^4^ http://www.arb-silva.de/

^5^ http://qiime.org/scripts/assign_taxonomy.html

^6^ https://unite.ut.ee/
