# Supplementary material for: The responses to long-term nitrogen addition of soil bacterial, fungal, and archaeal communities in a desert ecosystem
Source: Front Microbiol. 2022 Oct 13;13:1015588. doi: 10.3389/fmicb.2022.1015588 (PMC9606763; doi:10.3389/fmicb.2022.1015588)
Supplement: Supplementary file 1 [file Data_Sheet_1.docx]

**Supplementary Material**

**1.1 Supplementary Figures**

| 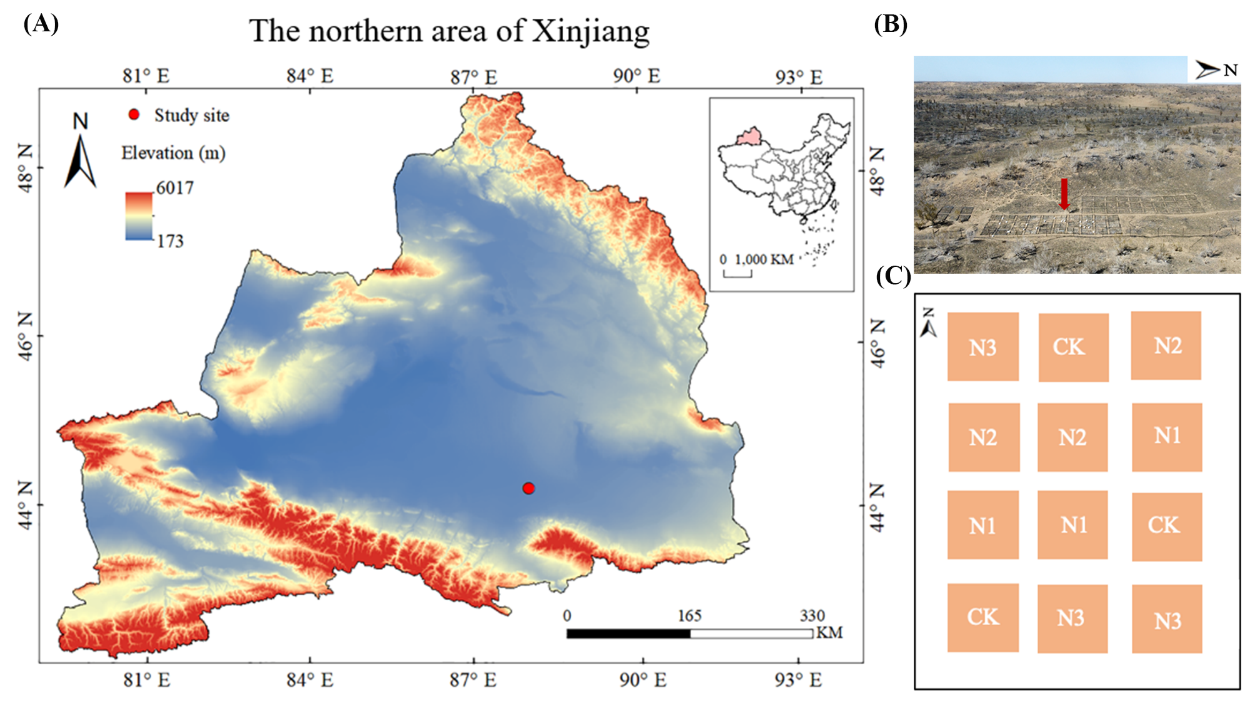**Supplementary Figure 1 \|** Geographical location of the study area. **(A)** The location of the study site. **(B)** The overlook diagram of the study site. **(C)** Diagram of the quadrat. |
| --- |

**2.1 Supplementary tables**

**Supplementary Table 1 |** Mantel test analysis for the correlation between soil properties and soil microbial community structure (OTUs level)

| **Microbial species** |  | **pH** | **SOC** | **TN** | **NO_3_^-^-N** | **NH_4_^+^-N** | **AP** |
| --- | --- | --- | --- | --- | --- | --- | --- |
| Bacteria | r | 0.1091 | 0.2937 | 0.1254 | 0.1063 | 0.0476 | 0.0847 |
|  | p | 0.2280 | 0.0650 | 0.1720 | 0.6910 | 0.3540 | 0.7110 |
| Fungi | r | 0.3916 | 0.1302 | 0.0287 | 0.1376 | 0.1385 | 0.0819 |
|  | p | **0.0190** | 0.7400 | 0.3890 | 0.7730 | 0.1620 | 0.7040 |
| Archaea | r | 0.3444 | 0.2592 | 0.1477 | 0.3561 | 0.2585 | 0.0804 |
|  | p | **0.0380** | 0.1380 | 0.8850 | 0.9930 | 0.9680 | 0.6800 |

The r-value indicates the correlation degree of the matrix. The p-value indicates the significance degree. SOC: soil organic carbon; TN: total nitrogen; NH_4_^+^-N: ammonium nitrogen; NO_3_^-^-N: nitrate nitrogen; AP: available phosphorus. Bold numbers indicate p < 0.05.
